# Accuracy of Machine Learning in Detecting Pediatric Epileptic Seizures: Systematic Review and Meta-Analysis

**DOI:** 10.2196/55986

**Published:** 2024-12-11

**Authors:** Zhuan Zou, Bin Chen, Dongqiong Xiao, Fajuan Tang, Xihong Li

**Affiliations:** 1 Department of Emergency West China Second University Hospital Sichuan University Chengdu China; 2 Key Laboratory of Birth Defects and Related Diseases of Women and Children Ministry of Education Sichuan University Chengdu China

**Keywords:** epileptic seizures, machine learning, deep learning, electroencephalogram, EEG, children, pediatrics, epilepsy, detection

## Abstract

**Background:**

Real-time monitoring of pediatric epileptic seizures poses a significant challenge in clinical practice. In recent years, machine learning (ML) has attracted substantial attention from researchers for diagnosing and treating neurological diseases, leading to its application for detecting pediatric epileptic seizures. However, systematic evidence substantiating its feasibility remains limited.

**Objective:**

This systematic review aimed to consolidate the existing evidence regarding the effectiveness of ML in monitoring pediatric epileptic seizures with an effort to provide an evidence-based foundation for the development and enhancement of intelligent tools in the future.

**Methods:**

We conducted a systematic search of the PubMed, Cochrane, Embase, and Web of Science databases for original studies focused on the detection of pediatric epileptic seizures using ML, with a cutoff date of August 27, 2023. The risk of bias in eligible studies was assessed using the QUADAS-2 (Quality Assessment of Diagnostic Accuracy Studies–2). Meta-analyses were performed to evaluate the C-index and the diagnostic 4-grid table, using a bivariate mixed-effects model for the latter. We also examined publication bias for the C-index by using funnel plots and the Egger test.

**Results:**

This systematic review included 28 original studies, with 15 studies on ML and 13 on deep learning (DL). All these models were based on electroencephalography data of children. The pooled C-index, sensitivity, specificity, and accuracy of ML in the training set were 0.76 (95% CI 0.69-0.82), 0.77 (95% CI 0.73-0.80), 0.74 (95% CI 0.70-0.77), and 0.75 (95% CI 0.72-0.77), respectively. In the validation set, the pooled C-index, sensitivity, specificity, and accuracy of ML were 0.73 (95% CI 0.67-0.79), 0.88 (95% CI 0.83-0.91), 0.83 (95% CI 0.71-0.90), and 0.78 (95% CI 0.73-0.82), respectively. Meanwhile, the pooled C-index of DL in the validation set was 0.91 (95% CI 0.88-0.94), with sensitivity, specificity, and accuracy being 0.89 (95% CI 0.85-0.91), 0.91 (95% CI 0.88-0.93), and 0.89 (95% CI 0.86-0.92), respectively.

**Conclusions:**

Our systematic review demonstrates promising accuracy of artificial intelligence methods in epilepsy detection. DL appears to offer higher detection accuracy than ML. These findings support the development of DL-based early-warning tools in future research.

**Trial Registration:**

PROSPERO CRD42023467260; https://www.crd.york.ac.uk/prospero/display_record.php?ID=CRD42023467260

## Introduction

Epilepsy is defined as a transient sign or symptom arising from abnormal, excessive, or synchronous neuronal activity in the brain [1]. It is the second most common neurological disorder [2]. Over 65 million people worldwide suffer from this mental disorder, equating to 1 in every 26 individuals [3]. In pediatric intensive care units, the prevalence of epileptic seizures among all hospitalized children is estimated to be 0.8% [4]. Hence, research into the prediction of epileptic seizures is particularly imperative.

Electroencephalography (EEG) has been established as an electrographic recording technique of brain activity, capable of timely predicting the occurrence of epileptic seizures from scalp EEG signals. This allows for more proactive and effective intervention for patients, making it an effective tool for the evaluation and diagnosis of epilepsy [5]. EEG is currently the gold standard for diagnosing neonatal epilepsy [6]. However, the interpretation of EEG primarily relies on the clinician’s previous experience, which can impact the interpretation of some critical signals. Therefore, monitoring epilepsy, especially in real time, remains a challenging task [7].

In the current epileptic seizure research, there is a lack of in-depth understanding of the mechanisms of epileptic seizures and accurate predictive models. Current research is still exploring the biological and neurological mechanisms of epileptic seizures, and there is a lack of models that can accurately predict and assess individual risk. In this study, most of the data came from publicly available datasets, including but not limited to the Children’s Hospital Boston-Massachusetts Institute of Technology (CHB-MIT) [8] and the University of Helsinki [9], which used the international 10-20 system configuration for the 19-channel EEG systems with a sampling rate of 256 Hz. With the rapid development of computer systems and ongoing advancements in statistical theories, artificial intelligence (AI) has gradually demonstrated a significant applicative value in clinical practice. This is particularly evident in the diagnosis and risk stratification of prognosis for some refractory diseases. In recent years, deep learning (DL) with its advantage of automatic feature extraction from images, has been extensively applied in image processing to assist in the auxiliary diagnosis of a wide variety of diseases.

For instance, Wei et al [10] conducted a study using machine learning (ML) to predict depression and anxiety of epilepsy patients in China, and Yossofzai et al [11] developed and validated an ML model to predict the outcome of epilepsy surgery in children. ML encompasses conventional ML and DL methods. In the past decade, DL has been used as a promising alternative to traditional ML and has been widely applied in various research fields. Truong et al [12] applied convolutional neural networks (CNNs) to different EEG datasets and demonstrated the effectiveness of DL. Daoud and Bayoumi [13] used cellular neural networks to extract meaningful features and then used recursive neural networks to classify them. Ozcan and Erturk [14] constructed 3D patterns based on the electrode positions and applied an image-based 3D CNN to predict epileptic seizures. Using directed transfer functions to explore the special information exchange between brain electrical channels, and then using cellular neural networks to predict epilepsy seizures, achieving satisfactory performance [15]. Yang et al [16] proposed a dual self-attention residual network to classify the short-time Fourier transform features of brain electrical signals.

In this context, multiple studies have also attempted to construct ML models for forecasting epileptic seizures based on different modeling variables, even DL models based on EEG to differentiate epileptic seizures. To date, both ML and DL lack a systematic understanding of their predictive accuracy. As such, this study aims to discern the accuracy of ML methods, including classical ML and DL models, in detecting seizures in children, and provide evidence-based recommendations for the development of AI in this field.

## Methods

### Study Registration

Our systematic review was implemented following the PRISMA (Preferred Reporting Items for Systematic Reviews and Meta-Analyses) 2020 guidelines [17] in Multimedia Appendix 1 and prospectively registered with PROSPERO (ID CRD42023467260).

### Eligibility Criteria

We developed detailed inclusion and exclusion criteria for our systematic review based on population, modeling, study type, language, and outcome measures, as detailed in Textbox 1.

**The inclusion and exclusion criteria.**

**Inclusion criteria**

- Population
  - Minor
- Modeling
  - Comprehensive construction of machine learning (ML) models for predicting epileptic seizures, including both traditional ML and deep learning approaches
- Study type
  - Case-control, cohort, nested case-control, cross-sectional studies
- Language
  - Studies published in English
- Outcome measures
  - Receiver operating characteristic, C-index, sensitivity, specificity, accuracy, recall, precision, confusion matrix,*F*_1_-score

**Exclusion criteria**

- Population
  - Studies that did not strictly distinguish between adults and minors
- Modeling
  - Studies focused solely on risk factors for epileptic seizures;
  - Studies that only assessed the predictive accuracy of single factors for seizures;
  - Studies focused solely on seizure image segmentation
- Study type
  - Reviews, guidelines, expert opinions, and non–peer-reviewed conference abstracts
- Language
  - Studies published in non-English languages
- Outcome measures
  - Studies missing any outcome measures assessing ML accuracy

### Data Sources and Search Strategy

PubMed, Cochrane, Embase, and Web of Science databases were thoroughly retrieved as of August 27, 2023. Both MeSH (Medical Subject Headings) terms and free-text keywords were used, without restrictions on publication region or year. Details of the search strategy are available in Table S1 in Multimedia Appendix 2.

### Study Selection and Data Extraction

All identified articles were imported into EndNote software (Clarivate). After deleting duplicates, titles and abstracts were screened to rule out irrelevant studies. The remaining articles were reviewed in full for inclusion. A spreadsheet was used to extract data, including title, first author, year of publication, type of study, patient origin, dataset source, number of epileptic seizure cases, total number of epileptic seizure cases in the training set, overfitting methods, number of cases in the validation set, missing value handling methods, variable selection, types of models used, and modeling variables.

In total, 2 independent investigators (ZZ and BC) implemented the literature screening and data extraction, followed by cross-checking. In case of any disagreements, a third investigator (DX) was consulted for resolution.

### Risk of Bias Assessment

The QUADAS-2 tool (Quality Assessment of Diagnostic Accuracy Studies–2) [18] was leveraged to appraise the risk of bias and applicability of the included studies. This tool evaluates 4 aspects, such as patient selection, index test, reference standard, and flow and timing. Each domain contains specific questions answered as “yes,” “no,” or “uncertain,” corresponding to a bias risk of “low,” “high,” or “uncertain,” respectively. Studies were considered at low risk of bias if all key questions in each domain were answered with “yes.” Any “no” response indicated potential bias, requiring evaluators to assess the risk level according to established guidelines. An “uncertain” rating signified insufficient information for a definitive judgment.

### Outcomes

This systematic review assessed diagnostic accuracy through sensitivity, specificity, positive likelihood ratio, negative likelihood ratio, diagnostic odds ratio, and the summary receiver operating characteristic curve. Calculating these estimates required diagnostic 4-fold tables. For ML, given the risk of overfitting, the diagnostic 4-fold tables in both training and validation sets were extracted from studies on ML. Funnel plots were used to analyze the publication bias of the C-index and then Egger test was performed for statistical evaluation of the publication bias. For DL, only the results of validation or test sets were considered.

### Statistical Analysis

Data analysis was executed using Stata 15.0 (StataCorp LLC). A bivariate mixed-effects model was used for the meta-analysis of sensitivity and specificity. However, if original studies lacked diagnostic 4-fold tables, they were calculated using two methods: (1) one based on sensitivity, specificity, precision, and case numbers, and (2) the other based on sensitivity and specificity calculated using the optimal Youden index and case numbers. Pooled estimates of sensitivity, specificity, likelihood ratios, diagnostic odds ratio, and their 95% CIs were calculated. The summary receiver operating characteristic curve area was also estimated. Publication bias assessment was conducted using funnel plots. Statistical significance was determined at *P*<.05. The studies included were all based on EEG-based ML methods, and we know that traditional ML and DL have certain differences in their image-processing capabilities and modeling efficiency. Therefore, to reduce heterogeneity, we conducted a subgroup analysis by model type for traditional ML and DL.

### Ethical Considerations and Consent to Participate

All analyses were based on previously published studies; thus, no ethical approval and patient consent are required.

## Results

### Study Selection

**Figure 1:**
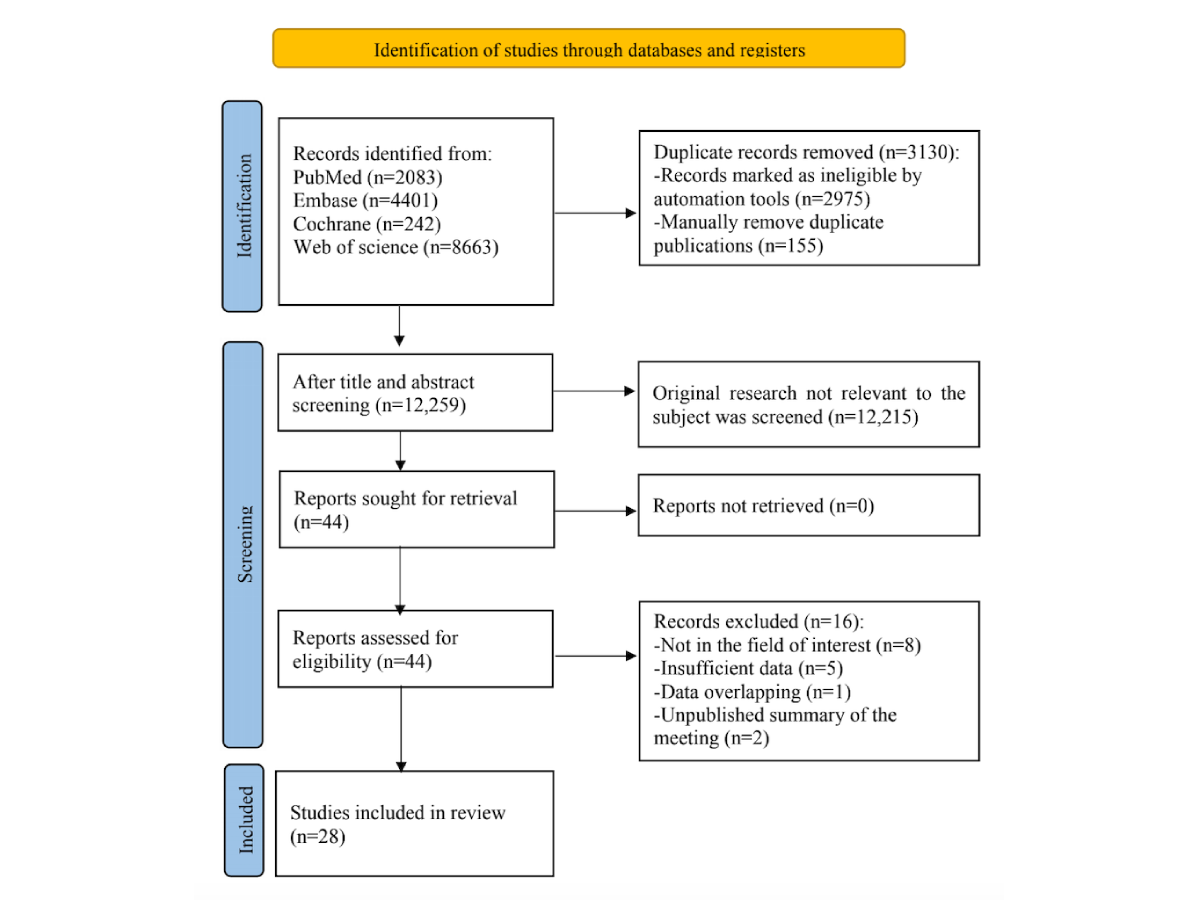
Literature selection flow chart.

Our systematic search identified 15,389 related articles, of which 3130 duplicates (2975 identified by software and 155 manually) were excluded. After title and abstract screening, we excluded 12,014 additional articles. Of the remaining 44 articles, 6 unpublished conference abstracts and 2 articles without full-text access were excluded. Among the remaining 36 articles, we excluded 5 for missing outcome measures, 1 for data overlap, and 2 for including adult participants. After these exclusions, 28 studies met the eligibility criteria [19-46]. Figure 1 illustrates the literature screening process.

### Study Characteristics

Table 1 presents the detailed characteristics of the 28 included studies, with 15 on ML [32-46] and 13 on DL [19-31]. Of these, 6 were multicenter studies [23,26,27,32,34,39], while the remaining 22 were single-center investigations. Furthermore, 5 studies were prospective [37,40,41,43,44], with the remaining 23 being retrospective. A total of 6 studies incorporated prospective external validations [26,27,30,40,43,44], 2 implemented multicenter external validations [26,27], and the remaining 22 used internal validations. Among studies with internal validation, 3 used 10-fold cross-validation [25,32,34], 4 used 5-fold cross-validation [21,35,37,41], 3 used the leave-one-out method [22,32,45], 1 used the Bootstrap method [33], and 10 used random sampling [19,20,23,28,31,36,38,39,42,46]. Of the total, 1 study did not specify the generation method of validation set [24]. Across the 28 studies, 5742 participants were included, with 1814 confirmed patients with epilepsy, ranging from 9 to 1117 patients per individual study. Furthermore, 10 studies were carried out in Asian countries, while 18 were conducted in Western countries. Out of total, 8 studies used EEG signals from the CHB-MIT dataset [19,22,24,31,34-36,39], another 8 used signals from The University of Helsinki dataset [20,21,23,25,28,29,38,42], and 3 used data from Cork University Maternity Hospital [26,27,45]. In addition, 1 ML study used 3 distinct models, each with separate training and validation sets [38]. All participants in the included studies were children, with 19 studies focusing specifically on neonates [19-21,23,25-30,32,33,36,38,42-46]. Studies on DL predominantly used CNN models. Of the 13 studies on DL detection of pediatric epileptic seizures included in this paper, 7 studies mainly applied the CNN model [21-23,25,27,29,30]. The DL models also included adaptive grey wolf optimizer (AGWO) [19], graph convolutional neural network [20], convolutional gated recurrent neural network [31], cross-feature fusion stream convolutional neural network [24], and pretrained deep convolution neural networks [28], and these models are mostly evolved from the CNN. ML studies used models including random forest (RF), k-nearest neighbors (KNN), support vector machine (SVM), extreme gradient boosting, adaptive boosting, and decision tree (DT). RF is an ensemble learning algorithm used for classification and regression [47], and 1 of its primary advantages is its robustness to noise. In our study, 4 original studies using this model were included [32,33,37,39]. KNN is the simplest ML tool with supervision for classification [48], and it depends on the predefined nearest number or the K value. One of the included original studies used this model [36]. DT predicts the correct classification by recursively partitioning the real space, and it consists of 2 types of multiple nodes, that is, leaf nodes and decision nodes. Of the total, 2 included original studies used this model [33,36]. SVM is an advanced algorithm mainly used for pattern recognition and feature reduction, using discriminative techniques to classify the input, and is best suited for binary classification [49]. Up to 5 original studies on ML in this study applied this model [34,38,42,44,45]. With the exception of 1 study that used electrocardiography as a variable [35], all other studies used EEG data. Clinical characteristics of patients were included as modeling variables in 6 studies [32,37,40,41,43,46].

**Table 1:** The detailed characteristics of the included studies.

No.	Author	Year	Country	Study design	Patient source	Task (prediction diagnosis)	Age	Dataset source	Events - P	Sample size - P	Training set - P	Verification mode	Validation set - P	Model classification	Modeling variable	Model classification (ML^a^ / DL^b^)
1	Pavel et al [32]	2023	Ireland	Retrospective cohort	Multicenter	Prediction	Neonatal	NCT02160171 and NCT02431780	53	162	162	10-fold cross-validation	None	RF^c^ and GBDT^d^	Clinical features + EEG^e^	ML
2	McKee et al [33]	2023	United States	Retrospective cohort	Single center	Prediction	Neonatal	Philadelphia, United States	150	1117	1117	Bootstrap	None	LR^f^, DT^g^, and RF	EEG	ML
3	Azriel et al [35]	2022	Israel	Retrospective cohort	Single center	Prediction	5.1 (0.8-11.5) y	CHB-MIT^h^	19	176	111	5-fold cross-validation	55	META^i^ + HRV^j^ + MOR^k^	ECG^l^	ML
4	Hu et al [37]	2021	United States	Prospective cohort	Single center	Prediction	<18 y	NCT03419260	184	719	719	5-fold cross-validation	None	RF	Clinical features + EEG	ML
5a	Elakkiya [38]	2021	India	Retrospective cohort	Single center	Diagnosis	Neonatal	The University of Helsinki	39	79	49	Random	13	SVM^m^	EEG	ML
5b	Elakkiya [38]	2021	India	Retrospective cohort	Single center	Diagnosis	Neonatal	The University of Helsinki	39	79	37	Random	25	ANN^n^	EEG	ML
5c	Elakkiya [38]	2021	India	Retrospective cohort	Single center	Diagnosis	Neonatal	The University of Helsinki	39	79	33	Random	25	CNN^o^	EEG	DL
6	Aayesha et al [39]	2020	Pakistan	Retrospective cohort	Multicenter	Diagnosis	1.5-22 y	CHB-MIT	23	23	23	Random	5	RF, SVM, KNN^p^, DT, and MLP^q^	EEG	ML
7	Fung et al [40]	2020	United States	Prospective cohort	Single center	Prediction	5.5 (1.43-13.2) y	NCT03419260	252	1033	719	External verification	314	LR	Clinical features + EEG	ML
8	Fung et al [41]	2020	United States	Prospective cohort	Single center	Prediction	5.5 (1.35-12.9) y	NCT03419260	184	719	719	5-fold cross-validation	719	LR	Clinical features + EEG	ML
9	Sansevere et al [43]	2020	United States	Prospective cohort	Single center	Prediction	Neonatal	Boston Children’s Hospital	73	210	210	External verification	None	LR	Clinical features + EEG	ML
10	Pan et al [44]	2020	China	Prospective cohort	Single center	Diagnosis	Neonatal	Peking University First Hospital	310	526	526	External verification	None	SVM, LR, AdaBoost^r^, XGBoost^s^, RF, and GBDT	EEG	ML
11	Temko et al [45]	2011	Ireland	Retrospective cohort	Single center	Diagnosis	Neonatal (term)	Cork University Maternity Hospital	17	55	55	the leave-one-out method	None	SVM	EEG	ML
12	Açikoğlu and Tuncer [42]	2019	Turkey	Retrospective cohort	Single center	Diagnosis	Neonatal	The University of Helsinki	39	79	79	Random	39	SVM	EEG	ML
13	Sethy et al [36]	2021	India	Retrospective cohort	Single center	Diagnosis	Neonatal	CHB-MIT	23	23	23	Random	23	DT, KNN, LDA^t^, LR. NB^u^, SVM	EEG	ML
14	Perez-Sanchez et al [34]	2022	Mexico	Retrospective cohort	Multicenter	Prediction	2-19 y	CHB-MIT	14	22	None	10-fold cross-validation	22	SVM and MLP	EEG	ML
15	Karayiannis et al [46]	2006	United States	Retrospective cohort	Single center	Diagnosis	Neonatal	The Clinical Research Centers for Neonatal Seizures	9	9	None	Random	9	QNNs^v^ and FFNN^w^	Clinical features + EEG	ML
16	Jaishankar et al [19]	2023	India	Retrospective cohort	Single center	Prediction	Neonatal	CHB-MIT	23	23	None	Random	8	AGWO^x^	EEG	DL
17	Raeisi et al [20]	2022	Italy	Retrospective cohort	Single center	Diagnosis	Neonatal	The University of Helsinki	39	79	None	None	79	GCNN^y^	EEG	DL
18	Gramacki A and Gramacki J [21]	2022	Poland	Retrospective cohort	Single center	Prediction	Neonatal	The University of Helsinki	57	79	None	5-fold cross-validation	57	CNN	EEG	DL
19	Gao et al [22]	2022	China	Retrospective cohort	Single center	Prediction	3-12 y	CHB-MIT	16	16	None	the leave-one-out method	16	CNN	EEG	DL
20	Borovac et al [23]	2022	Iceland	Retrospective cohort	Multicenter	Prediction	Neonatal	The University of Helsinki	38	79	79	Random	28	CNN	EEG	DL
21	Wang et al [24]	2021	China	Retrospective cohort	Single center	Prediction	9.89 y	CHB-MIT	23	23	None	none	23	CFS-CNN^z^	EEG	DL
22	Tanveer et al [25]	2021	Pakistan	Retrospective cohort	Single center	Prediction	Neonatal	The University of Helsinki	39	79	None	10-fold cross-validation	39	CNN	EEG	DL
23	O’Shea et al [26]	2021	Ireland	Retrospective cohort	Multicenter	Prediction	Preterm infants (GA<32 w)	CUMH^aa^, Parma University Hospital	23	33	17	External verification	10	DL	EEG	DL
24	Daly et al [27]	2021	Ireland	Retrospective cohort	Multicenter	Diagnosis	Neonatal	CUMH, the University of Helsinki	80	229	72	External verification	78	CNN	EEG	DL
25	Caliskan and Rencuzogullari [28]	2021	Turkey	Retrospective cohort	Single center	Diagnosis	Neonatal	The University of Helsinki	39	79	None	Random	79	p-DCNN^ab^	EEG	DL
26	Frassineti et al [29]	2020	Italy	Retrospective cohort	Single center	Diagnosis	Neonatal	The University of Helsinki	39	79	None	the leave-one-out method	39	CNN	EEG	DL
27	Ansari et al [30]	2019	Netherlands	Retrospective cohort	Single center	Diagnosis	Neonatal (term)	Sophia Children’s Hospital	48	48	26	External verification	22	CNN	EEG	DL
28	Affes et al [31]	2019	Tunisia	Retrospective cohort	Single center	Diagnosis	1.5-22 y	CHB-MIT	23	23	None	Random	13	CGRNN ^ac^	EEG	DL

^a^ML: machine learning.

^b^DL: deep learning.

^c^RF: random forest.

^d^GBDT: gradient boosting decision tree.

^e^EEG: electroencephalography.

^f^LR: logistic regression.

^g^DT: decision tree.

^h^CHB-MIT: Children’s Hospital Boston-Massachusetts Institute of Technology.

^i^META: metadata.

^j^HRV: heart rate variability.

^k^MOR: morphological features.

^l^ECG: electrocardiography.

^m^SVM: support vector machine.

^n^ANN: artificial neural network.

^o^CNN: convolutional neural networks.

^p^KNN: k-nearest neighbors.

^q^MLP: multilayer perceptron.

^r^AdaBoost: adaptive boosting.

^s^XGBoost: extreme gradient boosting.

^t^LDA: linear discriminant analysis.

^u^NB: naive Bayes.

^v^QNNs: Quantum Neural Net.

^w^FFNN: feedforward neural network.

^x^AGWO: adaptive grey wolf optimizer.

^y^GCNN: graph convolutional neural network.

^z^CFS-CNN: the cross-feature fusion stream convolutional neural network.

^aa^CUMH: Cork University Maternity Hospital.

^ab^p-DCNN: pretrained deep convolution neural networks.

^ac^CGRNN: convolutional gated recurrent neural network.

### Risk of Bias in Studies

**Figure 2:**
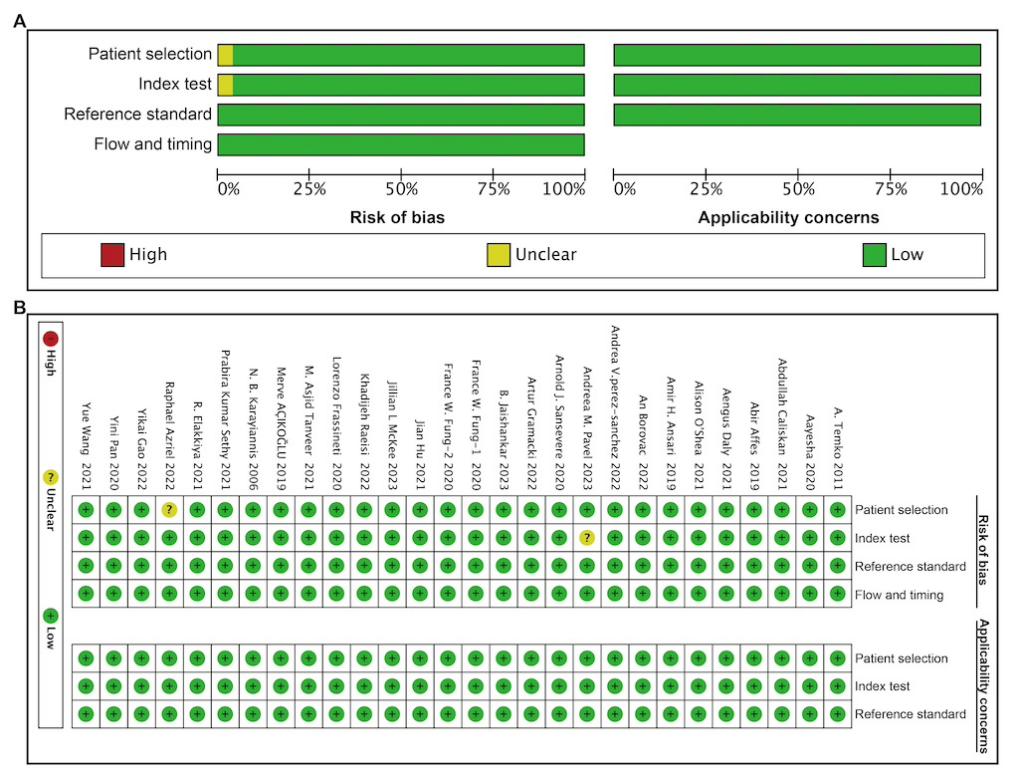
Risk of bias (A) graph and (B) summary.

In the 28 included studies, all cases were consecutive case series, eliminating concerns of case selection bias. Notably, the type of study design had minimal influence on the results of image-based DL studies [21,26,27], leading to a low risk of bias for this category. Conversely, conventional ML methods often involve manual segmenting regions of interest segmentation and extracting texture features, potentially introducing significant bias in case-control studies. Consequently, this study was categorized as having an unclear risk of bias [35]. There was a study [32] in which we doubted its results were interpreted without the outcome of the gold standard trial of epileptic seizures, the risk of bias of the study was classified as unclear. Further details are shown in Figure 2.

### Meta-Analysis

#### Machine Learning

**Figure 3:**
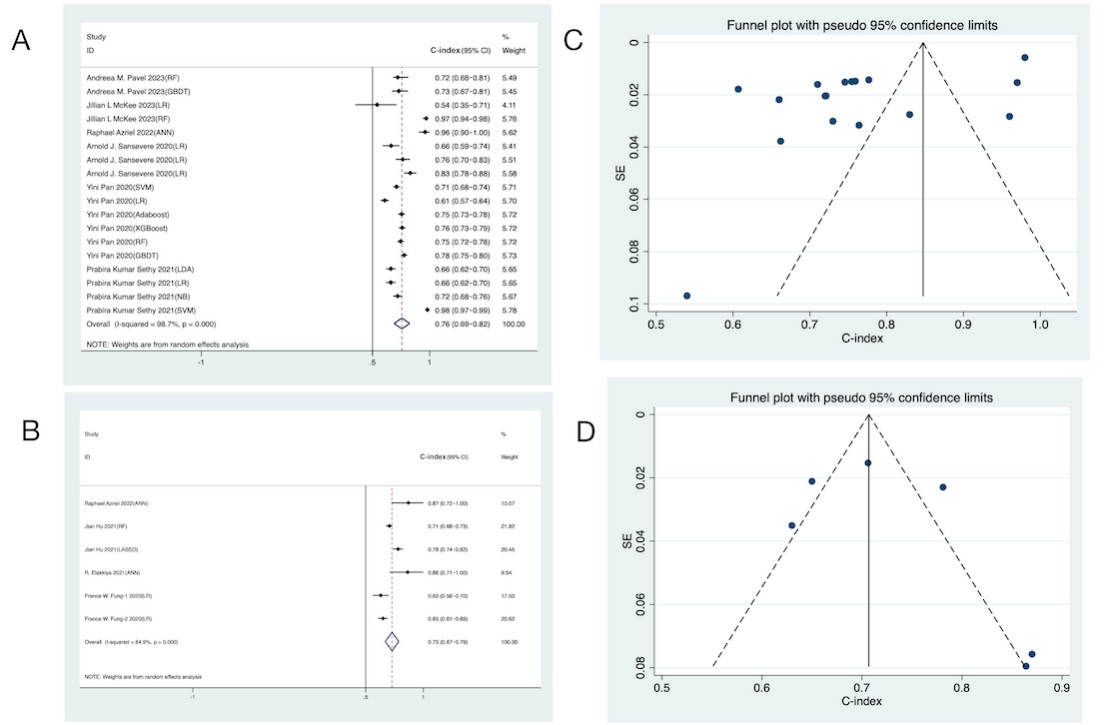
Forest and funnel plots of machine learning models for detecting seizures in children. The presence of repeated authors in the literature arises from the development of multiple machine learning models. (A) Forest plot illustrating the C-index summarization for the training set. (B) Forest plot illustrating the C-index summarization for the validation set. (C) Funnel plot illustrating the C-index for training set. (D) Funnel plot illustrating the C-index for the validation set. AdaBoost: adaptive boosting; ANN: artificial neural network; GBDT: gradient boosting decision tree; LDA: linear discriminant analysis; LR: logistic regression; NB: naive Bayes; RF: random forest; SVM: support vector machine; XGBoost: extreme gradient boosting.

The 15 studies on ML used multi-arm predictive diagnostic experiments, generating 25 training sets and 13 independent validation sets. A total of 18 training sets and 6 validation sets provided C-index values. A random-effects model revealed pooled C-indexes of 0.76 (95% CI 0.69-0.82) and 0.73 (95% CI 0.67-0.79) in training and validation sets, respectively (Figures 3A and 3B). The funnel plots of the pooled training and pooled validation sets of ML were analyzed, respectively. The funnel plot generated from the training set suggested an uneven but symmetric distribution of the C-indexes of the included studies, and Egger test indicated a possible publication bias (*P*=.001 and *P*<.05). On the other hand, the funnel plot from the validation set showed that distribution of the C-indexes tended to be symmetrical, and Egger test suggested no significant publication bias (*P*=.47 and *P*>.05; Figures 3C and 3D).

**Figure 4:**
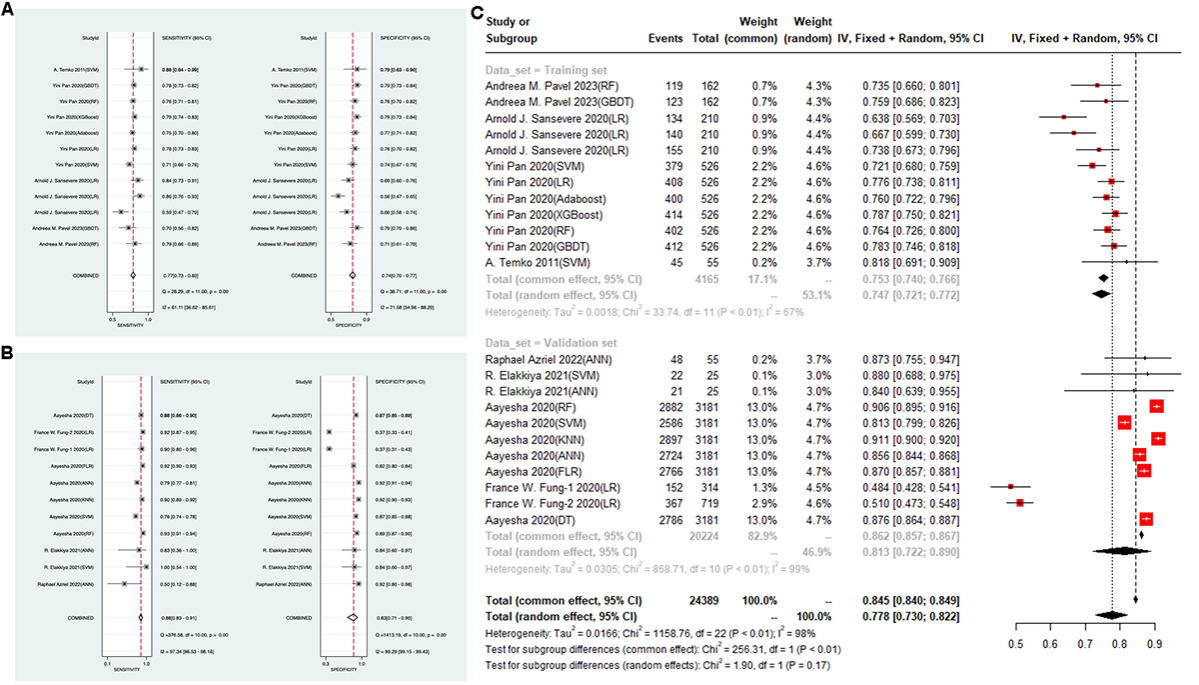
The forest plot shows the sensitivity, specificity, and accuracy of machine learning models in detecting seizures in children. The presence of repeated authors in the literature arises from the development of multiple machine learning models. (A) Sensitivity and specificity of the training set, (B) Sensitivity and specificity of the validation set, and (C) Accuracy of the machine learning models for both the training set and the validation set post summarization. AdaBoost: adaptive boosting; ANN: artificial neural network; DT: decision tree; GBDT: gradient boosting decision tree; KNN: k-nearest neighbors; LR: logistic regression; SVM: support vector machine; RF: random forest; XGBoost: extreme gradient boosting.

In addition, 12 training sets and 11 validation sets provided sensitivity, specificity, and accuracy. A random-effects model was leveraged. The analysis showed a pooled sensitivity of 0.77 (95% CI 0.73-0.80) and specificity of 0.74 (95% CI 0.70-0.77) for training sets (Figure 4A). For validation sets, the pooled sensitivity was 0.88 (95% CI 0.83-0.91) and specificity was 0.83 (95% CI 0.71-0.90; Figure 4B). The accuracy for the pooled training set and validation set in ML were 0.75 (95% CI 0.72-0.77) and 0.78 (95% CI 0.73-0.82), respectively (Figure 4C). No significant publication bias was detected in these results.

#### Deep Learning

**Figure 5:**
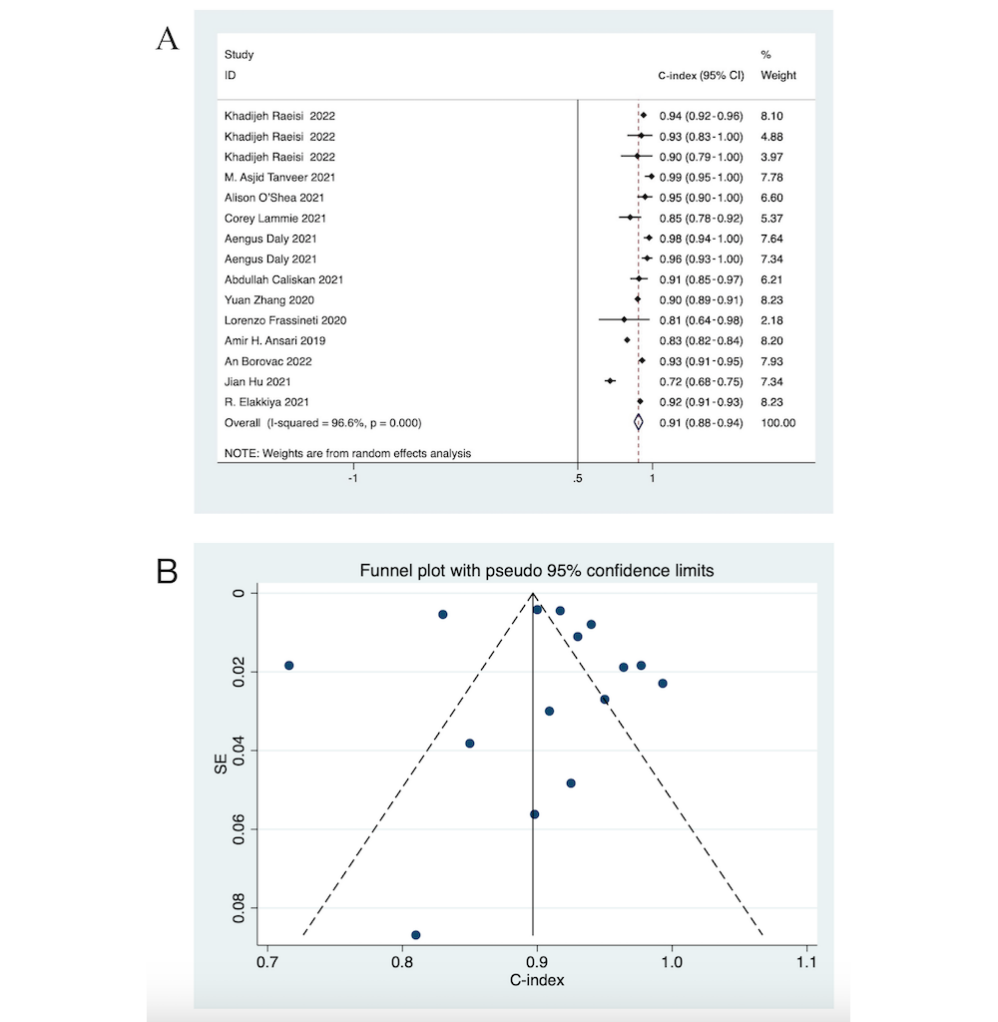
Forest and funnel plots of the C-index for the validation set for deep learning models for detecting seizures in children. The presence of repeated authors in the literature arises from the development of multiple deep learning models. (A) Forest plot illustrating the C-index summarization for the validation set. (B) Funnel plot illustrating the C-index for the validation set.

In total, 13 DL studies focused on DL for diagnosing pediatric epileptic seizures. Since DL is less susceptible to overfitting, only 26 validation sets were analyzed. Among the validation sets, 4 provided only the C-index, 10 provided only sensitivity, specificity, and accuracy without the C-index value, and the remaining 11 validation sets provided both the C-index as well as sensitivity, specificity, and accuracy. Therefore, a total of 15 validation sets were included in the C-index analysis for DL, while 21 validation sets were included in the analysis of sensitivity, specificity, and accuracy for DL. Of these, 15 validation sets provided C-index values. A random-effects model revealed a pooled C-index of 0.91 (95% CI 0.88-0.94; Figure 5A). The funnel plot of the C-indexes in the DL validation set also showed a generally symmetric distribution, and Egger test suggested no significant publication bias (*P*=.75 and *P*>.05; Figure 5B).

**Figure 6:**
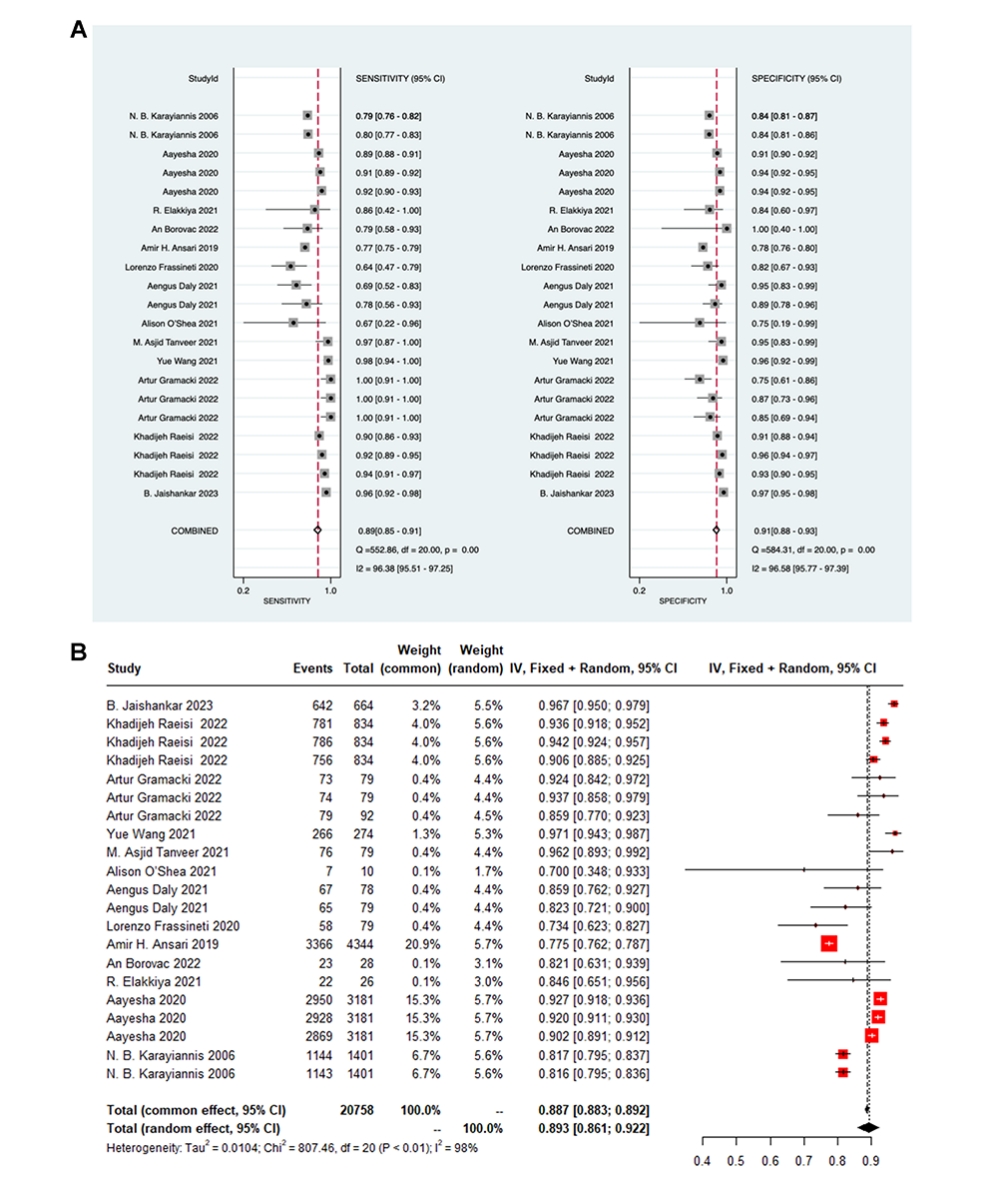
The forest plot shows the sensitivity, specificity, and accuracy of deep learning models in detecting seizures in children. The presence of repeated authors in the literature arises from the development of multiple deep learning models. (A) The application of deep learning for seizure detection in children demonstrates the sensitivity and specificity of ensemble methods and forest plots; (B) Accuracy of the deep learning validation set post summarization.

Furthermore, 21 validation sets provided sensitivity, specificity, or precision values. A random-effects model was leveraged for data analysis. The analysis showed a pooled sensitivity of 0.89 (95% CI 0.85-0.91), specificity of 0.91 (95% CI 0.88-0.93; Figure 6A), and an accuracy rate of 0.89 (95% CI 0.86-0.92; Figure 6B). Similarly, no significant publication bias was observed in these results.

## Discussion

### Summary of Main Findings

Real-time detection of epileptic seizures has profound clinical significance. Studies have shown that if a child, who has a normal nervous system without a history of neurological diseases, experienced unprovoked epileptic seizures without obvious acute causes, then the risk of recurrence is about 25% within the following 1 year, and 45%-50% within the following 3 years [50-52]. In this study, the results showed that AI-based methods have high sensitivity, specificity, and accuracy in detecting epileptic seizures. The overall C-index, sensitivity, and specificity of ML are 0.73 (95% CI 0.67-0.79), 0.88 (95% CI 0.83-0.91), and 0.83 (95% CI 0.71-0.90) in the validation set, respectively. DL exhibited even higher accuracy, with an overall C-index, sensitivity, and specificity of 0.91 (95% CI 0.88-0.94), 0.89 (95% CI 0.85-0.91), and 0.91 (95% CI 0.88-0.93) in the validation set. It was also found that EEG-based DL appears capable of detecting SE in a timely manner. This suggests that this study has the potential to be used in the development of portable devices for real-time monitoring in future research. Such devices could enhance the timely detection of epileptic seizures, enabling prompt clinical intervention and thereby reducing the risk of recurrence.

### Comparison With Other Reviews

Several noninvasive methods for epilepsy detection are actively investigated. In recent years, researchers have also explored other noninvasive means for detecting epileptic seizures besides EEG, such as magnetoencephalography (MEG). MEG measures the magnetic fields generated by the brain to provide information about neural activity during epileptic seizures. Similar to EEG, MEG can also be used to detect signal changes before epileptic seizures. Some studies have shown that MEG has specific advantages in detecting extratemporal epileptic spikes, especially those from epileptic lesions located on the surface of the brain [53-55]. A meta-analysis by Brændholt and Jensen [56] showed that the sensitivity and specificity of MEG for detecting epileptic seizures were 0.77 (95% CI 0.60-0.90) and 0.75 (95% CI 0.53-0.90), respectively. While this finding supports MEG as an accurate method for detecting SE, compared with the results of this study, it suggested that both EEG-based ML and DL may offer superior performance in detecting SE. Another method is electrical source imaging (ESI), which is a model-based imaging technique that integrates the spatial and temporal components of EEG to identify the sources of abnormal electrical activity related to epileptic seizures [57]. In a study by Ricci et al [58], the application of ESI in pediatric patients with refractory epilepsy was evaluated. The results showed that the sensitivity, specificity, and accuracy of ESI in predicting seizures in children were 0.57 (95% CI 0.34-0.78), 0.86 (95% CI 0.57-0.98), and 0.69 (95% CI 0.51-0.84), respectively (*P*=.01). Furthermore, 1 study showed that the diagnostic performance of ESI is comparable with that of magnetic resonance imaging and positron emission computed tomography. The sensitivity of these 3 methods for predicting epileptic seizures was 0.88, 0.71, and 0.66, respectively, while their specificity was 0.47, 0.71, and 0.59, respectively [59]. While these detection methods have shown promise, real-time monitoring of epileptic seizures remains a challenge, requiring further attention in clinical practice. Our study demonstrates that DL-based approaches offer promising results with a sensitivity of 0.89 (95% CI 0.85-0.91) and a specificity of 0.91 (95% CI 0.88-0.93) for pediatric epileptic seizure detection. Integrating DL based on EEG or electrocardiography into portable smart devices could significantly enhance real-time monitoring capabilities.

We have also noticed that there are permanent differences between DL and ML, and both have their own advantages. For ML, interpretable clinical features and corresponding ML models can be developed. For example, one of the original studies we included based on ML showed that the RF, SVM, and KNN models had a sensitivity of 0.93, 0.76, and 0.90, a specificity of 0.89, 0.76, and 0.90, and an accuracy of 0.91, 0.81, and 0.91 in predicting epileptic seizures, respectively [39]. Interpretable models in the clinical setting are still a direction that we are very interested in. However, for certain imaging applications, ML requires manual preprocessing, like segmenting regions of interest and extracting features. During this process, it is difficult to avoid any heterogeneity or bias due to manual experience, therefore, for image analysis, we prefer to use the DL to intelligently train the differences between them to develop intelligent detection tools. The primary advantage of CNN is that it can automatically detect important robust features without any manual intervention. The point is to estimate and determine the number of layers and the size of the filters in each layer, and the depth of the structure is the key to deep CNN. The network depth change will affect the convolutional receptive field and the corresponding learning feature complexity [60]. For instance, one of the original studies based on DL showed that the model had a sensitivity of 0.96, a specificity of 0.97, and an accuracy of 0.97 in predicting epileptic seizures, and was compared with common ML methods, such as RF, DT, and SVM [19].

In clinical practice, we need real-time monitoring for some diseases, such as epileptic seizures. However, ML still faces serious challenges in real-time disease monitoring, as the ML modeling is based on manual encoding, making it difficult to process images intelligently. Compared with ML, DL has significant advantages in real-time monitoring. We also found that researchers have done further work in other fields, developing wearable devices for real-time disease monitoring based on DL methods, such as monitoring heart-related diseases [61,62] or automatically assessing the severity of knee osteoarthritis [63].

### Advantages and Limitations

We found that current meta-analyses related to epilepsy detection are usually based on the direct diagnosis by clinical neurologists through the reading of EEG signals [64,65]. This study is the first study to discuss the use of AI-based methods, including ML and DL, for the prediction and diagnosis of epileptic seizures. Particularly, DL has shown very desirable accuracy in the diagnosis of SE, which may provide some theoretical support for the subsequent development of intelligent reading tools or wearable devices. However, several limitations should be considered. First, the number of cases used for model construction is limited, whereas ML algorithms require a larger sample size to build robust models. This may influence the generalizability of these results. Second, some included studies lack independent validation. Third, the detection performance of DL across different age groups remains uncertain. Third, we only searched for literature published in English in this study. For this, there are 2 primary reasons, one is there are certain search barriers in this study and the other is that considering most readers would need to review the relevant original studies while reading this article, we developed this search strategy to improve the readability for the readers. Of course, it is also a limitation of this study, and we hope that future research can cover more detection tools developed by diverse populations from different countries and ethnicities to validate the results of this study.

### Conclusions

Our systematic review demonstrates promising accuracy of AI methods in epilepsy detection. DL appears to offer higher detection performance compared with ML, this finding supports our initiative to further research and develop early warning tools using DL.

## Appendix

Multimedia Appendix 1 PRISMA (Preferred Reporting Items for Systematic Reviews and Meta-Analyses) 2020 checklist.

Multimedia Appendix 2 Literature search strategy.

## Abbreviations

AI: artificial intelligence
CHB-MIT: Children’s Hospital Boston-Massachusetts Institute of Technology
CNN: convolutional neural network
DL: deep learning
DT: decision tree
EEG: electroencephalography
ESI: electrical source imaging
KNN: k-nearest neighbors
MEG: magnetoencephalography
MeSH: Medical Subject Headings
ML: machine learning
PRISMA: Preferred Reporting Items for Systematic Reviews and Meta-Analyses
QUADAS-2: Quality Assessment of Diagnostic Accuracy Studies–2
RF: random forest
SVM: support vector machine

## Data Availability

All data generated or analyzed during this study are included in the article and supplementary material.
