# Supplementary material for: Accuracy of Machine Learning in Detecting Pediatric Epileptic Seizures: Systematic Review and Meta-Analysis
Source: J Med Internet Res. 2024 Dec 11;26:e55986. doi: 10.2196/55986 (PMC11669868; doi:10.2196/55986)
Supplement: Multimedia Appendix 2 [file jmir_v26i1e55986_app2.docx]

# Table S1

**1.Pubmed**

| Search number | Query | Results |
| --- | --- | --- |
| #1 | "Seizures"[Mesh] | 73,892 |
| #2 | (((((Seizures[Title/Abstract]) OR (Seizure[Title/Abstract])) OR (Non-Epileptic Convulsion[Title/Abstract])) OR (Non-Epileptic Convulsions[Title/Abstract])) OR (Convulsions[Title/Abstract])) OR (Convulsion[Title/Abstract]) | 159,417 |
| #3 | ((((((Seizures[Title/Abstract]) OR (Seizure[Title/Abstract])) OR (Non-Epileptic Convulsion[Title/Abstract])) OR (Non-Epileptic Convulsions[Title/Abstract])) OR (Convulsions[Title/Abstract])) OR (Convulsion[Title/Abstract])) OR ("Seizures"[Mesh]) | 177,459 |
| #4 | "Machine Learning"[Mesh] | 59,322 |
| #5 | (((((((((((((((((((((((((((machine learning[Title/Abstract]) OR (Transfer Learning[Title/Abstract])) OR (Deep learning[Title/Abstract])) OR (Ensemble Learning[Title/Abstract])) OR (artificial intelligence[Title/Abstract])) OR (random forest[Title/Abstract])) OR (neural network[Title/Abstract])) OR (neural networks[Title/Abstract])) OR (K-Nearest Neighbor[Title/Abstract])) OR (CNN[Title/Abstract])) OR (AlexNet[Title/Abstract])) OR (VGGNet[Title/Abstract])) OR (ResNet[Title/Abstract])) OR (GoogLeNet[Title/Abstract])) OR (Support vector machine[Title/Abstract])) OR (SVM[Title/Abstract])) OR (Gradient Boosting Machine[Title/Abstract])) OR (Nomogram[Title/Abstract])) OR (XGBoost[Title/Abstract])) OR (Adaboost[Title/Abstract])) OR (Decision tree[Title/Abstract])) OR (Naive Bayesian[Title/Abstract])) OR (Multilayer perceptron[Title/Abstract])) OR (Bayesian network[Title/Abstract])) OR (Radiomics[Title/Abstract])) OR (Radiomic[Title/Abstract])) OR (Prediction model[Title/Abstract])) OR (Risk model[Title/Abstract]) | 298,391 |
| #6 | ("Machine Learning"[Mesh]) OR ((((((((((((((((((((((((((((machine learning[Title/Abstract]) OR (Transfer Learning[Title/Abstract])) OR (Deep learning[Title/Abstract])) OR (Ensemble Learning[Title/Abstract])) OR (artificial intelligence[Title/Abstract])) OR (random forest[Title/Abstract])) OR (neural network[Title/Abstract])) OR (neural networks[Title/Abstract])) OR (K-Nearest Neighbor[Title/Abstract])) OR (CNN[Title/Abstract])) OR (AlexNet[Title/Abstract])) OR (VGGNet[Title/Abstract])) OR (ResNet[Title/Abstract])) OR (GoogLeNet[Title/Abstract])) OR (Support vector machine[Title/Abstract])) OR (SVM[Title/Abstract])) OR (Gradient Boosting Machine[Title/Abstract])) OR (Nomogram[Title/Abstract])) OR (XGBoost[Title/Abstract])) OR (Adaboost[Title/Abstract])) OR (Decision tree[Title/Abstract])) OR (Naive Bayesian[Title/Abstract])) OR (Multilayer perceptron[Title/Abstract])) OR (Bayesian network[Title/Abstract])) OR (Radiomics[Title/Abstract])) OR (Radiomic[Title/Abstract])) OR (Prediction model[Title/Abstract])) OR (Risk model[Title/Abstract])) | 303,471 |
| #7 | (((((((Seizures[Title/Abstract]) OR (Seizure[Title/Abstract])) OR (Non-Epileptic Convulsion[Title/Abstract])) OR (Non-Epileptic Convulsions[Title/Abstract])) OR (Convulsions[Title/Abstract])) OR (Convulsion[Title/Abstract])) OR ("Seizures"[Mesh])) AND (("Machine Learning"[Mesh]) OR ((((((((((((((((((((((((((((machine learning[Title/Abstract]) OR (Transfer Learning[Title/Abstract])) OR (Deep learning[Title/Abstract])) OR (Ensemble Learning[Title/Abstract])) OR (artificial intelligence[Title/Abstract])) OR (random forest[Title/Abstract])) OR (neural network[Title/Abstract])) OR (neural networks[Title/Abstract])) OR (K-Nearest Neighbor[Title/Abstract])) OR (CNN[Title/Abstract])) OR (AlexNet[Title/Abstract])) OR (VGGNet[Title/Abstract])) OR (ResNet[Title/Abstract])) OR (GoogLeNet[Title/Abstract])) OR (Support vector machine[Title/Abstract])) OR (SVM[Title/Abstract])) OR (Gradient Boosting Machine[Title/Abstract])) OR (Nomogram[Title/Abstract])) OR (XGBoost[Title/Abstract])) OR (Adaboost[Title/Abstract])) OR (Decision tree[Title/Abstract])) OR (Naive Bayesian[Title/Abstract])) OR (Multilayer perceptron[Title/Abstract])) OR (Bayesian network[Title/Abstract])) OR (Radiomics[Title/Abstract])) OR (Radiomic[Title/Abstract])) OR (Prediction model[Title/Abstract])) OR (Risk model[Title/Abstract]))) | 2,083 |

**2.Cochrane**

| Search number | Query | Results |
| --- | --- | --- |
| #1 | MesH descriptor: fSeizuresl explode all trees | 1504 |
| #2 | (Seizures):ti,ab,kw OR (Seizure):ti,ab,kw OR (Non-Epileptic Convulsion):ti,ab,kw OR (Non-Epileptic Convulsions):ti,ab,kw OR (Convulsions):ti,ab,kw | 10350 |
| #3 | (Convulsion):ti, ab, kw | 1093 |
| #4 | #1 or #2 or #3 | 10933 |
| #5 | MeSH descriptor: [Machine Learninal exolode all trees | 911 |
| #6 | (machine learninal:tiab kw OR (Transfer Learninal:ti ab.kw OR (Deep learnina):tiab.kw OR (Ensemble Learninal:ti ab.kw OR lartificial intellicencel:tiab.kw | 6368 |
| #7 | (random forest):ti.ab.kw OR (neural network):ti.ab.kw OR (neural networks):ti.ab.kw OR (K-Nearest Neighbor):ti.ab.kw OR (CNN):ti.ab.kw | 4148 |
| #8 | (AlexNet):ti.ab.kw OR (VGGNet):ti.ab.kw OR (ResNet):ti.ab.kw OR (GoogLeNet):ti.ab.kw OR (Support vector machine):ti.ab.kw | 550 |
| #9 | (SVM):ti.ab.kw OR (Gradient Boosting Machine):ti,ab,kw OR (Nomogram):ti,ab.kw OR (XGBoost):ti,ab.kw OR (Adaboost):ti.ab.kn | 1960 |
| #10 | (Decision tree):ti,ab.kw OR (Naive Bayesian):ti,ab,kw OR (Multilayer perceptron):tiab.kw OR (Bayesian network):ti,ab.kw OR (Radiomics):ti,ab.kn | 1795 |
| #11 | (Radiomic):ti,ab,kw OR (Prediction model): ti,ab,kw OR (Risk model): ti,ab,kw | 33204 |
| #12 | #5 or #6 or #7 or #8 or #9 or #10 or #11 | 42705 |
| #13 | #4 and #12 | 242 |

**3.Embase**

| Search number | Query | Results |
| --- | --- | --- |
| #1 | seizure'/exp | 229516 |
| #2 | seizure:ab,ti OR seizures:ab,ti OR 'non-epileptic convulsion':ab,ti OR 'non-epileptic convulsions':ab,ti OR convulsions:ab,ti OR convulsion:ab,ti | 237074 |
| #3 | #1 OR #2 | 314670 |
| #4 | machine learning'/exp | 410704 |
| #5 | machine learning':ab,ti OR 'transfer learning':ab,ti OR 'deep learning':ab,ti OR 'ensemble learning':ab,ti OR 'artificial intelligence':ab,ti OR 'random forest':ab,ti OR 'neural network':ab,ti OR 'neural networks':ab,ti OR 'k-nearest neighbor':ab,ti OR cnn:ab,ti OR alexnet:ab,ti OR vggnet:ab,ti OR resnet:ab,ti OR googlenet:ab,ti OR 'support vector machine':ab,ti OR svm:ab,ti OR 'gradient boosting machine':ab,ti OR nomogram:ab,ti OR xgboost:ab,ti OR adaboost:ab,ti OR 'decision tree':ab,ti OR 'naive bayesian':ab,ti OR 'multilayer perceptron':ab,ti OR 'bayesian network':ab,ti OR radiomics:ab,ti OR radiomic:ab,ti OR 'prediction model':ab,ti OR 'risk model':ab,ti | 347634 |
| #6 | #4 OR #5 | 557333 |
| #7 | #3 AND #6 | 4401 |

**4.Web of science**

| Search number | Query | Results |
| --- | --- | --- |
| #1 | Seizures (Topic) OR Seizure (Topic) OR Non-Epileptic Convulsion (Topic) OR Non-Epileptic Convulsions (Topic) OR Convulsions (Topic) OR Convulsion (Topic) | 173887 |
| #2 | machine learnin (Topic) OR Transfer Learning (Topic) OR Deep learning (Topic) OR Ensemble Learning (Topic) OR artificial intelligence (Topic) OR random forest (Topic) OR neural network (Topic) OR K-Nearest Neighbor (Topic) OR CNN (Topic) OR AlexNet (Topic) OR VGGNet (Topic) OR ResNet (Topic) OR GoogLeNet (Topic) OR Support vector machine (Topic) OR SVM (Topic) OR Gradient Boosting Machine (Topic) OR Nomogram (Topic) OR XGBoost (Topic) OR Adaboost (Topic) OR Decision tree (Topic) OR Naive Bayesian (Topic) OR Multilayer perceptron (Topic) OR Bayesian network (Topic) OR Radiomics (Topic) OR Radiomic (Topic) OR Prediction model (Topic) OR Risk model (Topic) | 2804812 |
| #3 | #1 AND #2 | 8663 |
